# A cross-sectional study on risk factors for infection with Parvovirus B19 and the association with anaemia in a febrile paediatric population in Ghana

**DOI:** 10.1038/s41598-020-72657-5

**Published:** 2020-09-24

**Authors:** Wiebke Herr, Ralf Krumkamp, Benedikt Hogan, Denise Dekker, Kennedy Gyau, Ellis Owusu-Dabo, Nimako Sarpong, Anna Jaeger, Wibke Loag, Doris Winter, Charity Wiafe Akenten, Daniel Eibach, Helmut Fickenscher, Anna Eis-Hübinger, Jürgen May, Benno Kreuels

**Affiliations:** 1 Department of Infectious Disease Epidemiology, Bernhard Nocht Institute for Tropical Medicine, 20359 Hamburg, Germany; 2 Kumasi Centre for Collaborative Research in Tropical Medicine, College of Health Sciences, Kwame Nkrumah University of Science and Technology, Kumasi, Ghana; 3 Institute for Infection Medicine, Christian-Albrecht University of Kiel, University Medical Center Schleswig-Holstein, 24105 Kiel, Germany; 4 University of Bonn Medical Center, Institute of Virology, 53127 Bonn, Germany; 5 Department of Tropical Medicine, Bernhard Nocht Institute for Tropical Medicine and I. Department of Medicine, University Medical Center Hamburg-Eppendorf, Martinistr. 52, 20246 Hamburg, Germany; 6 DZIF-German Center for Infection Research, Partnersite Hamburg-Lübeck-Borstel, 38124 Braunschweig, Germany

**Keywords:** Malaria, Viral infection, Paediatric research, Epidemiology

## Abstract

*Parvovirus B19* (B19V) occurs globally and can cause severe anaemia. The role of co-infections with *Plasmodium falciparum* (*P. falciparum)* has been controversially discussed. The study aimed to determine prevalence and severity of B19V infection, and the effect of co-infections on the risk for anaemia. Between November 2013 and April 2015 a total of 1186 hospital visits of children with fever admitted to a hospital in Ghana were recorded. Malaria, B19V and additional diagnostics for fever causes were performed. Recent B19V infection was defined as PCR and/or IgM positivity. Risk factors for a B19V infection and for anaemia were analysed. The prevalence of anaemia was compared between children with/without B19V infection, stratified for the presence of malaria. B19V IgM/PCR was positive in 6.4% (n = 76; 40 IgM + , 30 PCR + , 6 IgM + and PCR +). Among the B19V cases 60.5% had a simultaneous *P. falciparum* infection. B19V IgM positivity but not PCR positivity was associated with moderate-severe anaemia (OR = 2.6; 95%-CI: 1.3–5.3; *P* < 0.01 vs. OR = 0.9; 95%-CI: 0.4–1.8; *P* = 0.70). *P. falciparum* and IgM positive B19V infection were independent risk factors for anaemia with no evidence of effect modification. Our data show a significant association between B19V infection, defined as IgM but not PCR positivity, and moderate-severe anaemia. A multiplicative effect of B19V and *P. falciparum* infection was not found.

## Introduction

Infections with *human parvovirus B19* (B19V) occur worldwide; seroprevalence data in paediatric populations in sub-Saharan Africa vary between 14% in Kenya (children < 6 years)^1^ and more than 90% in a Nigerian population (children ≤ 2 years)^2^. The virus mainly leads to asymptomatic infection and primarily in children can cause erythema infectiosum, a self-limiting viral exanthema. B19V infection can also result in severe anaemia, especially in immunocompromised individuals and patients with underlying erythropoietic disorders^3^. B19V can induce cell apoptosis and inhibition of erythropoiesis, lasting on average ten days through targeting of the erythroid progenitor cells, i.e. the burst forming unit erythroid (BFU-E) and colony forming unit erythroid (CFU-E)^4,5^. While healthy individuals can compensate these temporary changes due to the long lifespan of erythrocytes^6^, vulnerable subgroups can suffer from serious illness as transient aplastic crisis and chronic anaemia^7^.

One such risk group is children in malaria-endemic areas. In this population, *P. falciparum*-malaria itself leads to anaemia via different pathogenetic mechanisms. First, intraerythrocytic parasite-growth causes acute haemolysis and decreased cell flexibility, followed by increased removal by the reticuloendothelial system in the spleen^8^. This effect can also be observed in non-affected erythrocytes^9^. In the course of the disease, especially in recurrent infection, hypersplenism may develop, which reinforces this selection mechanism. Second, autoimmune haemolytic factors can increase anaemia^8^. Lastly, cytokine responses mediate malfunctioning erythropoiesis, leading to a lack of reticulocytes and general dyserythropoiesis^9^.

Research on B19V infection in children from tropical areas has focused on anaemic study populations with contradictory conclusions about frequencies and clinical significance. In studies from Papua New Guinea, Kenya, Ghana and Tanzania B19V infection was described as a major risk factor for the development of anaemia^1,5,10–12^. A study from Niger rather suggests a temporary significance during viral outbreaks^2^. In contrast data from Malawi do not show any correlation between anaemia and the B19V infection^13^. Similarly, conflicting data have been published about the influence of co-infections of B19V and *P. falciparum* on the development and frequency of anaemia^1,11,14^.

The aim of this study was to determine the prevalence of B19V infection, its association with anaemia, and the effect of B19V co-infection with the malaria parasite *P. falciparum* in febrile hospitalized children.

## Methods

### Patients and clinical procedures

Between November 2013 and April 2015 all children with fever (tympanic temperature ≥ 38.0 °C) between four weeks and ≤ 15 years of age, admitted to Agogo Presbyterian Hospital in Ghana were prospectively recruited. Repeated inclusion was possible at least 30 days after initial recruitment. If a child presented twice with a positive B19V PCR, only the first visit was included in the analysis as we aimed at investigating acute B19V infections.

Depending on the presented symptoms blood, stool, urine, respiratory and/or cerebrospinal fluid samples were collected and screened for parasitic, viral and bacterial infection by microscopy, culture or PCR. A detailed description of the diagnostic evaluation has previously been published^15^.

### Laboratory analysis and case definitions

Thin and thick blood films were examined for malaria parasites, counting parasite number and species determination. Clinical malaria due to *P. falciparum* was defined as a parasite-density of asexual *P. falciparum* stages > 12,000/µl and fever ≥ 38.0 °C. The density cut-off was introduced using data of a control group from the study area in order to avoid overdiagnosis^15,16^.

For B19V diagnostics, serum collected on the day of admission was tested both by B19V qualitative real-time PCR and IgM- and IgG-antibody assays. The PCR followed an in-house protocol established under accreditation conditions according to EN-DIN-ISO-15189 at the Institute for Infection Medicine, University of Kiel, Germany. The sensitivity was 300 B19V genome copies/ml serum and the PCR primer and probe sequences targeted conserved sequences of all three genotypes. DNA was extracted with the RTP Pathogen Kit (STRATEC Molecular, Birkenfeld, Germany) from 100 µl serum after addition of lambda-phage DNA (Thermo Scientific SD0011, Waltham, MA, USA) as internal extraction and inhibition control. Nucleic acids were collected in 100 μl eluate. Five µl DNA eluate were added to 7.5 µl LC 480 Probe Master (Cat. No: 04707 494 001; Roche, Mannheim, Germany), 0.5 µl LC uracil-DNA glycosylase (Roche), and oligonucleotide primers and probes each at 1.0 pM concentration leading to total reaction volume of 15 μl. The PCR was performed on a Lightcycler 480 instrument (Roche) with the following amplification procedure: initially 40 °C for 10 min for uracil glycosylase treatment, the denaturation at 95 °C for 10 min followed by 45 amplification cycles of 95 °C for 10 s, 60 °C for 30 s, and terminating in 40 °C for 30 s. The B19V-specific primers and probe targeted the VP2 region: B19vp2F (TGG-CCC-ATT-TTC-AAG-GAA-GT), B19vp2r (CTG-AAG-TCA-TGC-TTG-GGT-ATT-TTT-C), and B19vp2TM (6FAM-CCG-GAA-GTT-CCC-GCT-TAC-AAC-BBQ). The internal lambda-phage DNA control was detected by the following primers and probe: Lambda-F (ATG-CCA-CGT-AAG-CGA-AAC-A), Lambda-R (GCA-TAA-ACG-AAG-CAG-TCG-AGT), and LAM-TM2 (YAK-ACC-TTA-CCG-AAA-TCG-GTA-CGG-ATA-CCG-BBQ). Each PCR was run together with a negative and positive control (TIB Molbiol, Berlin, Germany).

Serum samples were tested with IgM- and IgG-antibody assays (LIAISON Biotrin, Parvovirus B19 IgG, IgM ELISA, DiaSorin, Italy; recombinant VP2 expressed in baclovirus, qualitative assay). All IgM positive samples were retested with a second IgM-antibody assay (*recom*Well Parvovirus B19 IgM, Mikrogen, Neuried, Germany; recombinant VP1 and VP2, qualitative and quantitative assay). Only samples with positive results in both tests were considered positive for IgM.

Both, PCR or IgM positivity, were used as an indicator for recent B19V infection. For further analysis, B19V infections were divided into two groups (PCR positive and IgM positive). Six children were positive for both PCR and IgM and counted in both groups.

Blood count analysis on admission was performed with Sysmex KX-21N (Automated Hematology Analyzer, Sysmex Europe GmbH, Norderstedt, Germany). Anaemia cut-offs were used to differentiate between mild, moderate and severe anaemia among different age groups according to the World Health Organization (WHO) classification^17^ (Table 1). Anaemia was categorised into two groups: none or mild, and moderate to severe. This is in view of the limited clinical relevance of mild anaemia and the lack of association observed between B19V and mild anaemia (data not shown).

**Table 1:** Classification of anaemia.

Age	No anaemia Hb (g/dl)	Anaemia Hb (g/dl)		
		Mild	Moderate	Severe
1–59 months	≥ 11	10.0–10.9	7.0–9.9	< 7
5–11 years	≥ 11.5	11.0–11.4	8.0–10.9	< 8
12–14 years	≥ 12.0	11.0–11.9	8.0–10.9	< 8

### Statistical analysis

Statistical analysis were performed using STATA/SE v14 (StataCorp LLC, Texas, USA). Patient data were summarized using descriptive statistics. Categorical variables were displayed as percentages while continuous data were summarized as median and interquartile range (IQR). Comparisons of the median of non-parametric data among subgroups of the study were made using the median-test, as we anticipated low numbers of individuals in each group. Bivariate comparisons for categorical data were performed using the *Χ*^2^-Test and Fisher’s exact test as appropriate. Missing values were excluded from the analysis, which led to differing denominators in some analyses.

A socioeconomic score was constructed from nine indicator variables using principal component analysis (PCA) based on a tetrachoric correlation matrix. Subsequently, a binary variable on socioeconomic status was created by dividing the study group into two equally sized groups^18^. The nine indicator variables were: (1) membership in national health insurance, (2) subjective assessment of the financial situation of the family, (3) cooking inside/outside the house, (4) type of water supply, availability of (5) electricity and (6) window screens, ownership of (7) mobile phone, (8) television and (9) fridge.

To assess factors associated with B19V infection (defined as IgM or PCR positivity) a logistic regression analysis was performed. All variables with *P* ≤ 0.1 in the univariate model were included in a multivariate logistic regression model. Variables were then removed using a stepwise backwards selection method based on a likelihood-ratio test between the models including and excluding the respective variable, using *P* = 0.05 as the cut-off.

To detect a possible association between B19V infection and anaemia univariate logistic regression analysis was performed, distinguishing between the three subgroups of B19V IgM positive, B19V PCR positive and B19V IgM or PCR positive cases. Other factors putatively associated with anaemia (sex, age, siblings, ethnicity, socioeconomic status, glucose-6-phosphate dehydrogenase deficiency, sickle cell disease, *P. falciparum* infection) were assessed for an association in univariate logistic regression models. To adjust for possible confounding all variables were included in multivariate models with B19V IgM, B19V PCR and B19V IgM/PCR respectively. In addition all possible risk factors were analysed in uni- and multivariate linear regression models using the haemoglobin concentration (Hb) as a continuous variable as the outcome variable.

Possible effect-modification between B19V and *P. falciparum* was assessed by adding an interaction term to the full model. A likelihood-ratio test for interaction comparing the models was performed.

### Ethic approval

The Committee on Human Research, Publications and Ethics, School of Medical Science, Kwame Nkrumah University of Science and Technology, Kumasi, Ghana and the Ethics Committee of the Medical Association Hamburg, Germany approved the study. After informing about the study and procedures written informed consent was obtained from the guardian or parent prior to recruitment. The study was performed in accordance with all relevant national and international guidelines including the declaration of Helsinki.

## Results

### Study population

From November 2013 to April 2015, 1,238 hospital visits by children with fever (≥ 38.0 °C) were recruited to the study. Due to missing B19V PCR or serological results 49 visits were excluded. A further three visits were excluded as these children presented with positive B19V PCR for a second time. Therefore, a total of 1186 visits by 1108 children were included in the analysis. Of the 1108 children, 68 were recruited to the study more than once. The median age was two years (IQR 1–4 years), 54.4% of the children were male.

IgM was positive in 155/1186 (13.1%) participants by the Biotrin assay, of which a total of 46 samples (29.7%, 46/155) were confirmed positive using the Mikrogen assay. Of these 46 samples, six were also PCR positive. A further 30 samples were PCR positive alone, resulting in 76 samples (6.4%) positive for B19V in IgM and/or PCR. Among the 36 PCR positive samples, only three were IgG negative and six were positive for IgM and IgG.

Due to limited serum volume, not all negative samples could be tested with both assays. Results in a subset of initially (Biotrin assay) B19V IgM negative samples tested with both assays were in agreement in 99.2% (127/128).

Among patients with *P. falciparum* infection, more samples were IgM positive by the Biotrin assay (18.0% vs. 8.3%, *P* < 0.01). This was not the case for the Mikrogen assay (17.2% vs. 15.8%, *P* = 0.76). Index values of Biotrin assay and the associated Mikrogen result are displayed in the suppl. Figure 1. The proportion of confirmed positive results increased with higher sample to cut-off ratio (S/CO) values in the initial Biotrin assay result.

Among 76 children with B19V infection, 46 (60.5%) were co-infected with *P. falciparum* however in 92.1% (538/584) of children with *P. falciparum,* PCR and IgM were negative for B19V.

The majority of all B19V infections (67.1%, 51/76) occurred during the first eight months of recruitment, suggesting a cluster between November 2013 to June 2014 (suppl. Figures 2 and 3). Among children 13 months and older, B19V IgG-antibodies (Biotrin assay) were detected in 151 samples, resulting in a seroprevalence of 17.0% (151/889).

### Risk factors for B19V infection

Frequencies and associations of possible risk factors for B19V infection are shown in Table 2. Univariate logistic regression analysis showed weak evidence for an association between B19V infection and ethnicity, with a higher risk in children from ethnic groups originating in the North of Ghana (OR = 1.5; 95%-CI: 0.9–2.4; *P* = 0.09). Also children with a low socioeconomic status were more likely to be infected with B19V (OR = 2.0; 95%-CI: 1.2–3.3; *P* < 0.01), as well as children with siblings (1–2 siblings: OR = 2.0; 95%-CI: 0.9–4.2; 3–12 siblings: OR = 2.4; 95%-CI: 1.1–5.1; *P* = 0.05). There was no evidence for an association with B19V and sex, age, glucose-6-phosphate dehydrogenase deficiency or sickle cell disease. In multivariate analysis B19V infection only remained associated with a low socioeconomic status (OR = 2.1; 95%-CI: 1.3–3.4; *P* < 0.01).

**Table 2:** Risk factors for B19V infection.

	B19V infection (IgM + and/or PCR +)^a^	No B19V infection	Total	Univariate analysis Odds ratio (95%-CI)	*P*^b^	Multivariate analysis^c^ Odds ratio (95%-CI)	*P*^b^
Number of cases	76 (6.4)	1,110 (93.6)	1,186				
**Sex**							
Female	29 (38.2)	512 (46.1)	541 (45.6)	1			
Male	47 (61.8)	598 (53.9)	645 (54.4)	1.4 (0.9–2.2)	0.17	–	
**Age**							
Median in months (IQR)	37.5 (24–62.5)	29 (15–52)	29 (15–53)				
< 5 years	53 (69.7)	862 (77.7)	915 (77.1)	1			
≥ 5 years	23 (30.3)	248 (22.3)	271 (22.9)	1.5 (0.9–2.5)	0.12	–	
**Siblings**^d^							
None	9 (11.8)	251 (22.6)	260 (21.9)	1	0.05		
1–2	33 (43.4)	460 (41.4)	493 (41.6)	2.0 (0.9–4.2)			
3–12	33 (43.4)	384 (34.6)	417 (35.2)	2.4 (1.1–5.1)		–	
**Socioeconomic status**^d,e^							
High	26 (34.2)	560 (50.4)	586 (49.4)	1		1	
Low	50 (65.8)	534 (48.1)	584 (49.2)	2.0 (1.2–3.3)	< 0.01	2.1 (1.3–3.4)	< 0.01
**Ethnicity**^d^							
Akan	37 (48.7)	630 (56.8)	667 (56.2)	1			
Northeners	39 (51.3)	444 (40.0)	483 (40.7)	1.5 (0.9–2.4)	0.09	–	
**G6PD**							
No	75 (98.7)	1065 (95.9)	1140 (96.1)	1			
Yes	1 (1.3)	45 (4.1)	46 (3.9)	0.3 (0.0–2.6)	0.17	–	
**Sickle cell disease**							
No	73 (96.1)	1074 (96.8)	1147 (96.7)	1			
Yes	3 (3.9)	36 (3.2)	39 (3.3)	1.2 (0.4–4.1)	0.75	–	

^a^All samples with concordant positive serological results in Biotrin- and Mikrogen-assays were considered positive for IgM, all samples reactive by a qualitative PCR assay were considered PCR-positive. Six cases had a positive IgM and PCR result.

^b^Comparison for dichotomous or nominal data was performed using a logistic regression model.

^c^After stepwise backwards selection based on a likelihood ratio test between the models the socioeconomic status was the only remaining variable and risk factor.

^d^Missing data not listed (number of missing data for siblings and socioeconomic score n = 16, ethnicity n = 5; in addition 31 children belonged to other ethnicities).

^e^The socioeconomic score was constructed using nine indicator variables (membership of national health insurance, subjective assessment of the financial situation of the family, cooking inside/outside the house, type of water supply, availability of electricity and window screens, ownership of mobile phone, television and fridge) that were included into a tetrachoric correlation and principal component analysis (PCA). Subsequently, a binary variable on socioeconomic status was created in further risk factor analysis by dividing the population into two groups.

### Clinical presentation of B19V infection

There was no specific symptom that could be used to clinically diagnose a B19V infection. A rash only occurred in two patients (2.6%). As fever was an inclusion criterion to the study, all children presented with this symptom. Common symptoms in patients with B19V infection were vomiting (44.7%), cough (21.1%) and diarrhoea (17.1%). Children with B19V infection had a similar admission duration as children without markers of B19V infection (median = 2; IQR: 1–3 vs. median = 2; IQR: 2–4 days respectively; *P* = 0.01). There were no relevant differences in clinical presentation between the two groups of B19V infection (IgM positive and PCR positive, data not shown). Symptom frequencies and anaemia prevalence are summarized in Table 3.

**Table 3:** Clinical details of children with B19V infection.

	No B19V infection (%)	B19V infection (IgM and/or PCR +) (%)
Total	1110 (93.6)	76 (6.4)
Vomiting	437 (39.4)	34 (44.7)
Cough	285 (25.7)	16 (21.1)
Diarrhoea	189 (17.0)	13 (17.1)
Spleen enlargement	98 (8.8)^a^	10 (13.2)
Respiratory distress	106 (9.6)^a^	9 (11.8)
Liver enlargement	76 (6.9)^a^	7 (9.2)
Abdominal pain	56 (5.1)	4 (5.3)
Generalized lymphadenopathy	3 (0.3)^a^	2 (2.7)^a^
Rash	24 (2.2)^a^	2 (2.6)
Neurological symptoms	32 (2.9)	0
**Body temperature (°C)**		
Median (IQR)	39 (38.5–39.6)	39.3 (38.7–39.8)
**Duration of admission (days)**		
Median (IQR)	2 (2–4)	2 (1–3)
**Anaemia**^b^		
Severe	131 (11.8)	8 (10.5)
Moderate	419 (37.8)	40 (52.6)
Mild	195 (17.6)	10 (13.2)
No anaemia	364 (32.8)	17 (22.4)

^a^Missing data (No B19V infection: spleen enlargement n = 12, resp. distress n = 9, liver enlargement n = 11, gen. lymphadenopathy n = 5, rash n = 1; B19V infection: gen. lymphadenopathy n = 1).

^b^Hb-value available for 1184 cases.

### Risk factors for anaemia

Overall prevalence of anaemia in the study population was 67.8% (803/1184; two cases without Hb-value). Of these 17.3% (n = 139) had severe anaemia, 57.2% (n = 459) had moderate anaemia and 25.5% (n = 205) mild anaemia. For risk factor analysis cases of moderate and severe anaemia were compared to the group of children with mild and no anaemia. By univariate analysis, associations between moderate-severe anaemia were observed among children with sickle cell disease (OR = 4.7; 95%-CI: 2.0–10.7; *P* < 0.01), glucose-6-phosphate dehydrogenase deficiency (OR = 3.7; 95%-CI: 1.8–7.5; *P* < 0.01), children with low socioeconomic status (OR = 3.1; 95%-CI: 2.4–3.9; *P* < 0.01) and children from the north of Ghana relative to children from the Akan ethnicity (OR = 2.8; 95%-CI: 2.2–3.5; *P* < 0.01). Infection with *P. falciparum* increased risk for moderate-severe anaemia with an OR of 1.9 (95%-CI: 1.5–2.4; *P* < 0.01). These effect estimates changed slightly in the multivariate models, but the interpretation of the results remained similar (Table 4).

**Table 4:** Risk factors for anaemia, univariate and adjusted analysis.

	Cases with moderate and severe anaemia (%)	Univariate analysis Odds ratio (95%-CI)	*P*^a^	Model with B19V (PCR or IgM) Adjusted analysis^b^ Odds ratio (95%-CI)	*P*^a^	Model with B19V-IgM Adjusted analysis^b^ Odds ratio (95%-CI)	*P*^a^	Model with B19V-PCR Adjusted analysis^b^ Odds ratio (95%-CI)	*P*^a^
Total	598 (50.5)								
**Sex**									
Female	257 (47.6)	1		1		1		1	
Male	341 (52.9)	1.2 (1.0–1.6)	0.07	1.3 (1.0–1.6)	0.07	1.3 (1.0–1.6)	0.07	1.3 (1.0–1.6)	0.06
**Age**									
< 5 years	458 (50.2)	1		1		1		1	
≥ 5 years	140 (51.7)	1.1 (0.8–1.4)	0.70	1.1 (0.8–1.4)	0.73	1.1 (0.8–1.4)	0.71	1.1 (0.8–1.5)	0.70
**Siblings**									
None	119 (20.1)	1	0.20	1		1		1	
1–2	256 (43.2)	1.3 (0.9–1.7)		1.1 (0.8–1.5)	0.83	1.1 (0.8–1.5)	0.90	1.1 (0.8–1.5)	0.80
3–12	217 (36.7)	1.3 (1.0–1.8)		1.0 (0.7–1.5)		1.0 (0.7–1.4)		1.0 (0.7–1.5)	
**Ethnicity**									
Akan	265 (39.8)	1		1		1		1	
Northeners	312 (64.7)	2.8 (2.2–3.5)	< 0.01	1.8 (1.4–2.5)	< 0.01	1.8 (1.4–2.5)	< 0.01	1.8 (1.4–2.5)	< 0.01
**Socioeconomic status**^c^									
High	216 (36.9)	1		1		1		1	
Low	376 (64.5)	3.1 (2.4–3.9)	< 0.01	2.1 (1.5–2.8)	< 0.01	2.1 (1.5–2.8)	< 0.01	2.1 (1.6–2.8)	< 0.01
**G6PD**									
No	562 (49.4)	1		1		1		1	
Yes	36 (78.3)	3.7 (1.8–7.5)	< 0.01	2.4 (1.1–5.0)	0.03	2.3 (1.1–5.0)	0.03	2.3 (1.1–4.9)	0.03
**Sickle cell disease**									
No	566 (49.4)	1		1		1		1	
Yes	32 (82.1)	4.7 (2.0–10.7)	< 0.01	6.8 (2.9–16.3)	< 0.01	7.1 (3.0–17.0)	< 0.01	6.9 (2.9–16.6)	< 0.01
***P. falciparum***^d^									
No	256 (42.6)	1		1		1		1	
Yes	342 (58.7)	1.9 (1.5–2.4)	< 0.01	1.9 (1.4–2.4)	< 0.01	1.9 (1.5–2.4)	< 0.01	1.9 (1.5–2.4)	< 0.01
**Parvovirus B19**									
Negative	550 (49.6)	1		1		–		–	
IgM or PCR pos	48 (64.0)	1.8 (1.1–2.9)	0.02	1.5 (0.9–2.5)	0.14	–		–	
**IgM**^e^									
Negative	565 (49.7)	1		–		1		–	
Positive	33 (71.7)	2.6 (1.3–4.9)	< 0.01	–		2.6 (1.3–5.3)	< 0.01	–	
**PCR**^e^									
Negative	578 (50.3)	1		–		–		1	
Positive	20 (57.1)	1.3 (0.7–2.6)	0.43	–		–		0.9 (0.4–1.8)	0.70

^a^Comparison for dichotomous or nominal data was performed using a logistic regression model.

^b^Adjusted for all other variables in the table.

^c^The socioeconomic score was constructed using nine indicator variables (membership of national health insurance, subjective assessment of the financial situation of the family, cooking inside/outside the house, type of water supply, availability of electricity and window screens, ownership of mobile phone, television and fridge) that were included into a tetrachoric correlation and principal component analysis (PCA). Subsequently, a binary variable on socioeconomic status was created in further risk factor analysis by dividing the population into two groups.

^d^Clinical malaria with *P. falciparum* was defined as a parasite-density of *P. falciparum* > 12,000/µl and fever ≥ 38.0 °C.

^e^All samples with concordant positive serological results in Biotrin- and Mikrogen-assays were considered positive for IgM, all samples reactive by a qualitative PCR assay were considered PCR positive. Six cases had a positive IgM and PCR.

Among the children with B19V infection 64.0% (48/75) had moderate-severe anaemia. In univariate association the odds ratio for moderate-severe anaemia among those with any marker of B19V infection was of 1.8 (95%-CI: 1.1–2.9; *P* = 0.02), the association weakened in multivariate analysis (OR: 1.5; 95%-CI: 0.9–2.5; *P* = 0.14). More children with a positive IgM result had moderate-severe anaemia relative to children with a positive PCR result (71.7% vs. 57.1%). PCR positivity was not associated with a higher risk for moderate-severe anaemia (OR: 1.3; 95%-CI: 0.7–2.6; *P* = 0.43), while children who were IgM positive had an increased risk for moderate-severe anaemia (OR: 2.6, 95%-CI: 1.3–4.9; *P* < 0.01). In the multivariate analysis with adjustment for all parameters found to be associated with moderate-severe anaemia, IgM positivity and not PCR positivity remained associated with moderate-severe anaemia (IgM: OR: 2.6, 95%-CI: 1.3–5.3; *P* < 0.01; PCR: OR: 0.9; 95%-CI: 0.4–1.8; *P* = 0.70), Similarly, the median Hb-level was lower (9.0 g/dl vs. 10.1 g/dl; *P* = 0.08). Linear regression models using the Hb as a continuous variable led to similar results (suppl. Table 1).

### B19V, *P. falciparum* and anaemia

As illustrated above, both *P. falciparum* infection and B19V infection, defined as IgM positivity, were independent risk factors for moderate-severe anaemia while there was no evidence for an association between B19V PCR positivity and moderate-severe anaemia (Table 4). The likelihood-ratio-test between models with and without an interaction term between B19V and *P. falciparum* did not support the hypothesis of effect modification between the two (IgM: *P* = 0.91; PCR: *P* = 0.79).

## Discussion

Studies on the clinical relevance of B19V infection in malaria-endemic areas and the role of B19V and *P. falciparum* co-infections in severe anaemia show contradictory results^1,2,5,6,10–14^. In our study among febrile, hospitalized children in Ghana, an association was observed between moderate-severe anaemia and B19V IgM positivity but not with B19V PCR positivity. In addition, *P. falciparum* and IgM positive B19V infection were independent risk factors for moderate-severe anaemia with no evidence of effect modification.

B19V affects erythropoiesis by its replication in erythroid progenitor cells and thereby may cause anaemia^4^. While early studies on B19V infection and anaemia in malaria-endemic areas showed no association with anaemia^2,13^, more recent studies found the viral infection to be an important risk factor. In studies from Papua New Guinea and Ghana B19V infection was a major risk factor for anaemia^5,10,11^.

As the definition of B19V infection varies between studies, the comparison of the results is challenging. While Wildig et al*.* defined the B19V infection as presence of IgM and DNA^5^, Manning et al*.* required positivity of at least one of the two markers^10^ and Duedu et al*.* tested the samples only by PCR^11^. Toan et al*.* conducted serological assays only in PCR positive samples^14^ and Tizeba et al*.* performed only IgM and IgG testing^12^. Table 2 in the supplement provides an overview of the cited studies, their study populations, B19V definitions and performed laboratory analysis.

In contrast to our findings, Duedu et al*.* found a higher risk for severe anaemia in already mildly anaemic children if B19V PCR was positive (OR = 4.1; 95%-CI: 0.82–20.28) however the number of B19V infections was too low to draw robust conclusions^11^. Nevertheless our study accords with recently published data from Tanzania where an association between anaemia and B19V (defined as IgM positivity) was found^12^. Similarly, a study from Papua New Guinea found an association between severe anaemia and B19V infection in the subgroup of individuals with positive IgM, but not in the subgroup of children with positive PCR only^5^. In data from Kenya no association between severe anaemia and B19V IgM or IgG or DNA positivity was found, but an association between high IgM-levels and severe anaemia was shown^1^. The clear association of IgM positivity and anaemia would fit to the temporal coincidence of haemoglobin drop and high IgM levels during the natural course of B19V infection, when DNA-levels may already have dropped^1^. On the other hand, in some patients with B19V infection low amounts of B19V DNA may be detected in blood for years after acute infection^19,20^. In addition, remnant B19V DNA strands may cause positive PCR results^21^. Therefore a positive PCR result might not unequivocally indicate an acute infection leading to a possible underestimation of the risk for anaemia in B19V infections. In our population 15 children with positive B19V PCR did not have moderate or severe anaemia. These cases could represent persistent PCR positive results following past infection. In support of this, 33 of 36 PCR positive samples had additional IgG-antibodies, possibly indicating past infection. Concomitant IgG and IgM were identified in six PCR positive cases. Differentiation of active B19V infection and detection of B19V viral DNA indicating past infection is challenging.

Our study highlights diagnostic difficulties that may be common in several viral infections in sub-Saharan Africa. Commonly used PCRs may not detect the genotype 3 of B19V, that is characterized by higher genome diversity^22^ and predominantly found in sub-Saharan-Africa^20^. Our PCR was developed using all available information about genome diversity, but not previously validated in sub-Saharan-African populations. Therefore, non-detection cannot be fully excluded and could contribute to the low number of patients that were positive for both PCR and IgM in our study.

We found B19V and *P. falciparum* to be independent risk factors for anaemia, as described before^5^. Our data showed no effect modification between simultaneous B19V and *P. falciparum* infection. Previous data from Ghana described co-infections as a risk factor for severe anaemia with an OR of 2.2 (95%-CI: 0.4–12.5), however, the confidence interval around this estimate was wide due to a small sample size (6/234)^11^. Toan et al. found B19V to decrease hemoglobin levels in children with mild but not in children with severe *P. falciparum*-malaria. The authors did not provide explanations for this finding^14^. The most recent study from Tanzania described a difference of median Hb between small groups of children with co-infections (n = 12) and *P. falciparum* mono-infection (n = 34) (4.6 vs. 7.1 g/dl)^12^.

In our study 67.1% of B19V infections occurred during the first eight months of recruitment. The first publication on B19V infection reported a high prevalence in the first study period, followed by a rather low prevalence during the second observation time, suggesting an epidemic outbreak^2^. While Wildig et al*.* found no temporal clustering during the four years of sample collection in Papua New Guinea^5^, they described a increased prevalence of B19V IgM in one of five years of analysis in Kenya^1^. Seasonal occurrence might partly explain different findings and prevalence of B19V. Furthermore varying inclusion criteria of the studies (anaemia vs. *P. falciparum* infection vs. febrile infection) and age distribution in the study populations should be considered.

The role of laboratory diagnostics for acute B19V infection have been discussed extensively elsewhere^23,24^. The testing of both, serology and PCR, has been recommended, as each can be the only marker of recently acquired or acute infection^25^.

We found high discordancy between the IgM results obtained by the Biotrin and Mikrogen assays that has not been described before. Former systematic comparisons of these assays revealed no relevant differences^26,27^, but were performed in different settings. In our study, false positive IgM results could have been triggered by the polyclonal B-cell-stimulation during *P. falciparum* infection^28^. While 18.0% of the children with *P. falciparum* infection were IgM positive with the Biotrin assay, the percentage among children without *P. falciparum* infection was only 8.3% (*P* < 0.01). Also, false positive cross-reaction during measles, rubella virus, EBV or HSV infection or other infections have been described and cannot be ruled out in our study population^22^ but probably will not account for the great difference between the two assays. On the other hand the Mikrogen assay could be less sensitive in the studied setting, but existing literature provides no evidence of differing sensitivity^26,27^ and it is unclear how a lower sensitivity specifically in this setting could be explained.

In the cases of HCV infection in Africa, high rates of discordancy between serological and molecular assays have been described. The reason for this remains unclear (false positive serological results vs. non-detection of genotype differences of the RNA assay), but may indicate a high unspecific background activity in African populations leading to unreliability of some serological tests^29^.

The combination of PCR and serological testing is broadly established for the diagnosis of acute B19V infection^30^. However, we illustrated several aspects of diagnostic uncertainty. To improve B19V diagnostics the viral load can be determined by quantitative PCR and it may be possible to develop cut-off values suggestive of acute, past or rather persistent infection^31^. IgG-avidity-testing may also help to diagnose an acute infection^22^. Furthermore, our data indicate that the currently available commercial antibody-assays and PCR may not be sufficiently validated for testing in African population and further validation is warranted.

Due to our study design only children with fever at admission were included, while children with intake of antipyretics and therefore presentation without fever as well as anaemic children without fever were not included. This may have led to exclusion of children with B19V infection possibly with anaemia but no fever leading to an underestimation of the effect B19V has on the risk of anaemia. Due to limited resources iron deficiency, known as a major contributor to anaemia in children in low-income settings was not assessed. Probably all children were in an inflammatory status, therefore inflammation could not be studied as a risk factor for anaemia. Despite a large number of study participants only 76 children had a B19V infection resulting in smaller subgroups with anaemia and/or simultaneous *P. falciparum* infection, limiting the available power to perform subgroup analysis.

## Conclusion

Our data show a significant association between B19V infection and moderate-severe anaemia in febrile hospitalized children in Ghana. We can confirm the previously described significance of *P. falciparum* infection for anaemia in this context, while a multiplicative effect of B19V and *P. falciparum* infection could not be found. Prior to further investigation existing assays should be validated in sub-Saharan Africa, tested for sensitivity of detection of different viral genotypes and possible interaction in malaria-endemic areas.

## Supplementary information

Supplementary Information.

## Data Availability

The datasets generated and analysed during the current study are available from the corresponding author on reasonable request.
