# Supplementary material for: A cross-sectional study on risk factors for infection with Parvovirus B19 and the association with anaemia in a febrile paediatric population in Ghana
Source: Sci Rep. 2020 Sep 24;10:15695. doi: 10.1038/s41598-020-72657-5 (PMC7515863; doi:10.1038/s41598-020-72657-5)
Supplement: Supplementary file 1 — Supplementary Information. [file 41598_2020_72657_MOESM1_ESM.pdf]

A cross-sectional study on risk factors for infection with Parvovirus B19 and the association with anaemia in a febrile paediatric population in Ghana

Wiebke Herr, Ralf Krumkamp, Benedikt Hogan, Denise Dekker, Kennedy Gyau, Ellis Owusu-Dabo, Nimako Sarpong, Anna Jaeger, Wibke Loag, Doris Winter, Charity Wiafe Akenten, Daniel Eibach, Helmut Fickenscher, Anna Eis-Hübinger, Jürgen May, Benno Kreuels.

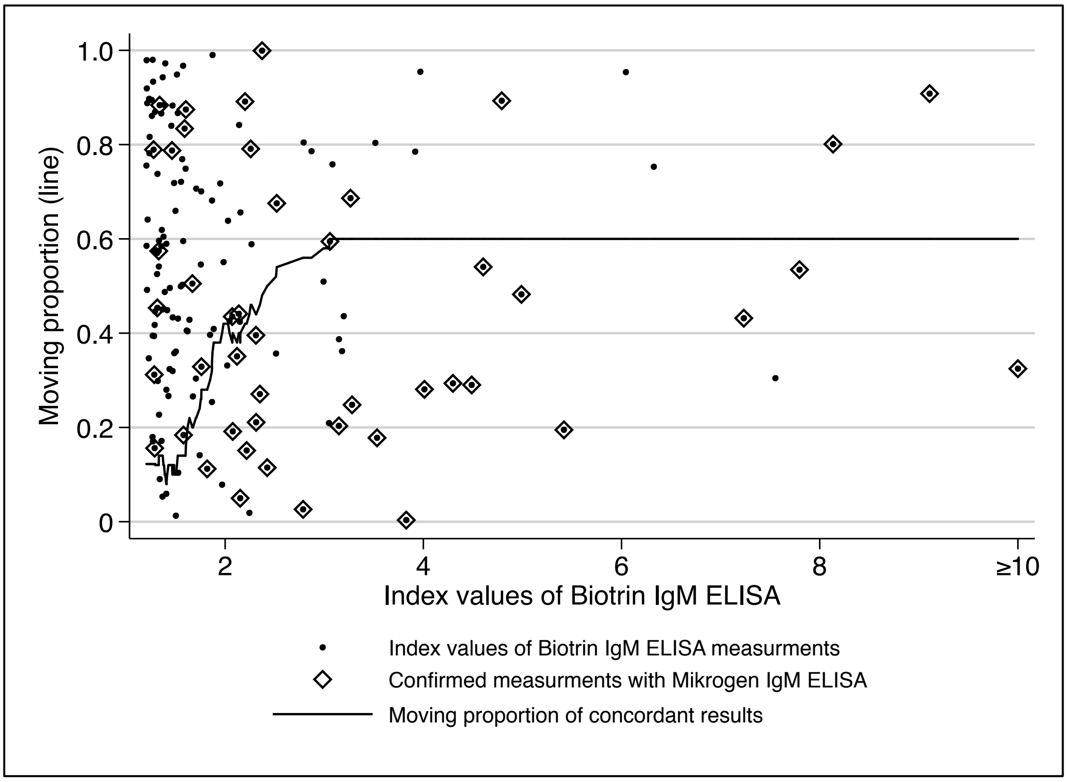

Supplement Figure 1. Overview of IgM-results of Biotrin- and Mikrogen-Testkits. Points and rectangles represent individual IgM-measurements (x-axis), which are randomly distributed along the y-axis. The line shows the proportion of positive Biotrin-IgM-results confirmed with the Mikrogen Testkit over a moving window of 50 patients, that are ordered according to their IgM-index-values.

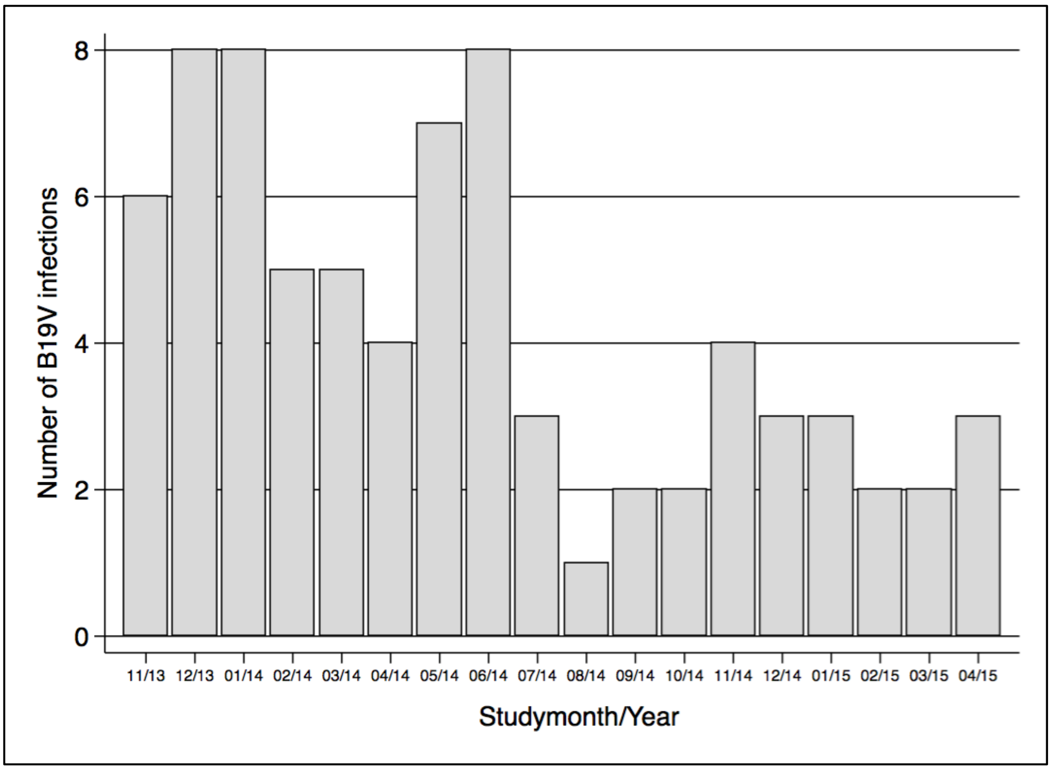

Supplement Figure 2. Temporal variability of B19V Infection. The figure shows the absolute number of cases with B19V infection (IgM or PCR positive) in each study month.

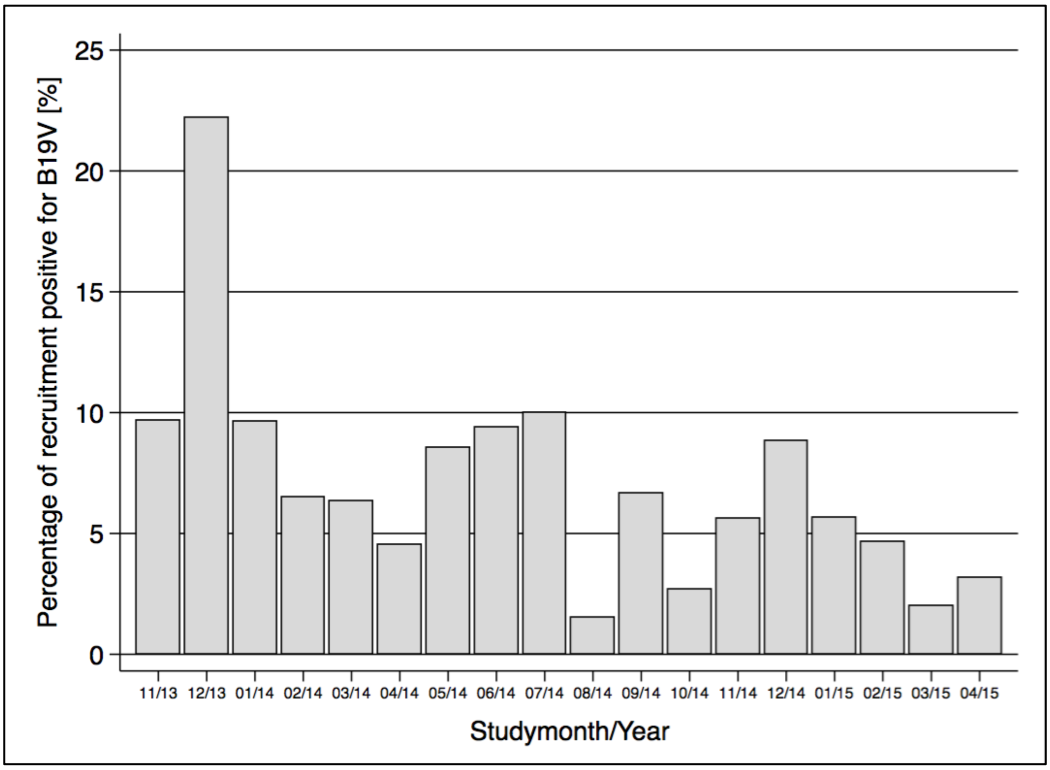

Supplement Figure 3. Relative frequency of B19V Infections. The figure displays the percentage of recruited cases per month tested positive for B19V infection (IgM or PCR positive)

Supplement Table 1. Risk factors for anaemia: Linear regression models using the Hb-value as a continuous variable.

	Univariate analysis: Coefficient (95%-CI); Hb [g/dl]	*P*[1]	Model with B19V (PCR or IgM) Multivariate analysis[2] Coefficient (95%-CI); Hb [g/dl][3]	*P*[1]	Model with B19V-IgM Multivariate analysis[2] Constant, Coefficient (95%-CI); Hb [g/dl][4]	*P*[1]	Model with B19V-PCR Multivariate analysis[2] Constant, Coefficient (95%-CI); Hb [g/dl][5]	*P*[1]
**Sex**								
**female**								
**male**	-0.27 (-0.53- -0.01)	0.04	-0.23 (-0.47–0.02)	0.07	-0.23 (-0.47–0.12)	0.06	-0.24 (-0.49- -0.00)	0.05
**Age**								
**< 5 years**								
**≥ 5 years**	0.80 (0.49–1.11)	<0.01	0.88 (0.58–1.18)	<0.01	0.9 (0.58–1.18)	<0.01	0.86 (0.56–1.16)	<0.01
**Siblings**								
**none**								
**1-2**	-0.14 (-0.49–0.21)		-0.14 (-0.49–0.21)		0.004 (-0.31–0.32)		-0.02 (-0.34–0.30)	
**3-12**	-0.10 (-0.46–0.56)	0.73	-0.02 (-0.19–0.14)	0.96	-0.03 (-0.37–0.30)	0.96	-0.06 (-0.40–0.28)	0.93
**Ethnicity**								
**Akan**								
**Northeners**	-1.28 (-1.54- -1.02)	<0.01	-0.63 (-0.92- -0.33)	<0.01	-0.63 (-0.92- -0.34)	<0.01	-0.63 (-0.92- -0.34)	<0.01
**Socioeconomic status**[6]								
**high**								
**low**	-1.38 (-1.63- -1.13)	<0.01	-0.88 (-1.17- -0.59)	<0.01	-0.87 (-1.18- -0.58)	<0.01	-0.91 (-1.20- -0.61)	<0.01
**G6PD**								
**no**								
**yes**	-1.63 (-2.30- -0.96)	<0.01	-0.95 (-1.56- -0.33)	<0.01	-0.96 (-1.58- -0.34)	<0.01	-0.90 (-1.52- -0.28)	<0.01
**Sickle cell disease**								
**no**								
**yes**	-2.36 (-3.08- -1.64)	<0.01	-2.82 (-3.50- -2.14)	<0.01	-2.87 (-3.55- -2.18)	<0.01	-2.86 (-3.54- -2.18)	<0.01
P. falciparum[7]								
**no**								
**yes**	-0.60 (-0.86- -0.34)	<0.01	-0.57 (-0.82- -0.32)	<0.01	-0.57 (-0.81- -0.32)	<0.01	-0.58 (-0.83- -0.34)	<0.01
**Parvovirus B19**								
**negative**								
**IgM or PCR pos.**	- 0.48 (-1.02–0.05)	0.08	-0.29 (-0.78–0.20)	0.25	-			
**IgM**[8]								
**negative**								
**positive**	-0.98 (-1.66- -0.31)	<0.01	-		-0.95 (-1.57- -0.34)	<0.01	-	
**PCR**[8]								
**negative**								
**positive**	0.18 (-0.59–0.95)	0.64	-		-	-	0.58 (-0.12–1.28)	0.11

[1] Comparison for dichotomous or nominal data was performed using a linear regression model.
[2] Adjusted for all other variables in the table.

[3] R^2: 0.195
[4] R^2: 0.200
[5] R^2: 0.196
[6] The socioeconomic score was constructed using nine indicator variables (membership of national health insurance, subjective assessment of the financial situation of the family, cooking inside/outside the house, type of water supply, availability of electricity and window screens, ownership of mobile phone, television and fridge) that were included into a tetrachoric correlation and principal component analysis (PCA). Subsequently, a binary variable on socioeconomic status was created in further risk factor analysis by dividing the population into two groups.
[7] Clinical malaria with *P. falciparum* was defined as a parasite-density of *P. falciparum* >12 000/µl and fever ≥38,0°C.
[8] All samples with concordant positive serological results in Biotrin- and Mikrogen-assays were considered positive for IgM, all samples reactive by a qualitative PCR assay were considered PCR positive. Six cases had a positive IgM and PCR.

Supplement Table 2. Overview of study populations, B19V definitions and used laboratory diagnostics.

	Laboratory diagnostics PCR	Antibody testing	Definition of B19V-infection	Study population	Definition of anaemia
Jones et al., 1990; Niger	In-situ hybridization (Anderson et al. 1985)	Radioimmunoassay (Cohen et al. 1983)	DNA hybridization or positive IgM status	120 Children in the outpatient department with packed cell volume (PCV)10-20%; 24 were studied in detail.	Severe anaemia: PCV <20%
Yeats, Daley, & Hardie, 1999; Malawi	Nested PCR	No	Positive PCR result	313 children with malaria attending hospital; comparison of children with severe anaemia and no anaemia	Severe anaemia: PCV ≤ 15%<br>No anaemia: PCV ≥30%
Wildig et al., 2006; Papua New Guinea	Nested PCR, primer design according to genomic sequence	EIA Biotrin, Ireland; tested in duplicate	Positive IgM and PCR result	169 children 6-59 months with severe anaemia,169 matched controls; children presented with presumptive malaria in health centers	Severe anaemia: haemoglobin ≤ 5 g/dl
Wildig et al., 2010; Kenya	PCR (primers according to Candotti et al., 2004); only 198 samples tested	EIA Biotrin, Ireland; tested in duplicate	Positive IgM	264 children under six years with severe anaemia, 263 matched controls; all hospitalized children	Severe anaemia: haemoglobin ≤ 5 g/dl
Manning et al., 2012; Papua New Guinea	PCR (primers according to Wildig et al. 2006)	EIA Biotrin, Ireland	Positive IgM or PCR result	143 children 0.5-10 years with severe anaemia admitted tot he hopsital; 120 healthy controls from community immunization clinic	Severe anaemia: haemoglobin ≤ 5 g/dl
Duedu et al., 2013 Accra	Semi-nested PCR (Parsyan et al.)	No	Positive PCR	234 hospitalized children (0-12 years)	Severe anaemia: haemoglobin ≤ 5 g/dl; For analysis formation of three groups (regardless of age): haemoglobin <8 g/dl; 8-10.9 g/dl; ≥ 11 g/dl
Toan et al., 2013 Gabun	Nested PCR (Toan et al. 2006) and quantitative PCR (Bultman et al. 2003, Tschope et al. 2005)	ELISA DxSelect FocusDiagnostic, Germany conducted only in subgroup	Positive PCR	197 children at the hospital with P.falciparum malaria, 85 healthy controls	Comparison of continous haematocrit levels,no categories
Tizeba et al, 2017, Tanzania	No	EIA DRG Instruments GmbH, Germany	Positive IgM status	265 hospitalized children < 5years	Severe anaemia <7 g/dl; moderate anaemia 7-9.9 g/dl; mild anaemia ≥ 10 g/dl
